# Quantifying bone marrow inflammatory edema in the spine and sacroiliac joints with thresholding

**DOI:** 10.1186/s12891-017-1861-1

**Published:** 2017-11-28

**Authors:** Ioanna Chronaiou, Ruth S. Thomsen, Else M. Huuse, Leslie R. Euceda, Susanne J. Pedersen, Mari Hoff, Beathe Sitter

**Affiliations:** 1 Department of Circulation and Medical Imaging, Faculty of Medicine and Health Sciences, NTNU – Norwegian University of Science and Technology, 7491 Trondheim, Norway, Trondheim, Norway; 2 Department of Public Health and Nursing, Faculty of Medicine and Health Sciences, NTNU – Norwegian University of Science and Technology, Trondheim, Norway; 3 Department of Radiology and Nuclear Medicine, St Olav’s Hospital, University Hospital in Trondheim, Trondheim, Norway; 4 Copenhagen Center for Arthritis Research, Center for Rheumatology and Spine Diseases, Rigshospitalet - Glostrup, Copenhagen, Denmark; 5 Department of Rheumatology, St Olav’s Hospital, University Hospital in Trondheim, Trondheim, Norway

**Keywords:** Psoriatic arthritis, MRI, Image processing, SPARCC, Bone marrow inflammatory edema

## Abstract

**Background:**

Psoriatic Arthritis (PsA) is a chronic inflammatory arthritis that develops in patients with psoriasis. Inflammatory edema in the spine may reflect subclinical disease activity and be a predictor of radiographic progression. A semi-quantitative method established by the spondyloarthritis research consortium of Canada (SPARCC) is commonly used to assess the disease activity in MR images of the spine. This study aims to evaluate thresholding for quantification of subtle bone marrow inflammation in the spine and the sacroiliac (SI) joints of patients with PsA and compare it with the SPARCC scoring system.

**Methods:**

Short tau inversion recovery (STIR) MR images of the spine (*N* = 85) and the SI joints (*N* = 95) of patients with PsA (*N* = 41) were analyzed. A threshold was applied to visible bone marrow in order to mask areas with higher signal intensity, which are consistent with inflammation. These areas were considered as inflammatory lesions. The volume and relative signal intensity of the lesions were calculated. Results from thresholding were compared to SPARCC scores using linear mixed-effects models. The specificity and sensitivity of thresholding were also calculated.

**Results:**

A significant positive correlation between the volumes and mean relative signal intensities, which were calculated by thresholding analysis, and the SPARCC scores was detected for both spine (*p* < 0.001) and SI joints (*p* < 0.001). For the spine, thresholding had sensitivity and specificity of 83% and 76% respectively, while for the SI joints the values were 51% and 88% respectively.

**Conclusions:**

Thresholding allows quantification of subtle bone marrow inflammatory edema in patients with psoriatic arthritis, and could support SPARCC scoring of the spine. Improved image processing and inclusion of automatic segmentation are required for thresholding of STIR images to become a rapid and reliable method for quantitative measures of inflammation.

**Trial registration:**

NCT02995460 (December 14, 2016) – Retrospectively registered.

## Background

Psoriatic Arthritis (PsA) is a chronic inflammatory joint disease associated with psoriasis [1] that manifests with inflammation in peripheral joints, axial skeleton, enthesitis and dactylitis [2]. Magnetic resonance imaging (MRI) allows visualization of inflammation and damage in all structures involved in PsA [3] and has been found to be more sensitive to inflammatory changes than clinical examination [4]. The prevalence of PsA ranges from 20 to 420 per 100,000 population in all countries except Japan, where prevalence is lower [5].

The prevalence of axial PsA varies from 25% to 75% of PsA patients depending on the criteria used [6, 7]. In a subgroup of patients with axial PsA, there is subclinical inflammation in the absence of clinical symptoms. Detecting radiographic involvement of the spine and the sacroiliac (SI) joints in these patients is important for diagnosis and classification. Accurate quantification of small inflammatory lesions in the spine and SI joints is important as it may reflect subclinical disease activity [3] and be a predictor of radiographic progression [8, 9]. Additionally, an accurate method that can detect minor changes will be able to assess the effect of treatment or intervention. A semi-quantitative method established by the spondyloarthritis research consortium of Canada (SPARCC) can be used in order to assess the disease activity in MR images of the spine and SI joints. This scoring method is reliable and sensitive to changes [10], but it requires a trained reader and is labor-intensive. A computer-aided and potentially automatic method for the quantification of bone marrow inflammation is thus a possible time-efficient alternative.

Manual methods for image analysis rely on human vision, which is very sensitive, but are reader-dependent and prone to subjective errors and variation. Automatic methods offer advantages over manual methods of analysis. They are standardized and reproducible and have a consistent accuracy. Moreover, automatic methods follow a systematic approach, thus are highly repeatable. Once established, the procedure can easily be consistently applied in a large number of images, is objective and less time-consuming.

Thresholding has been used in a previous study to quantify inflammation in the SI joints of patients with chronic lower back pain originating in the SI joints [11]. Application of this approach is based on the fact that inflammatory lesions have higher signal intensity than normal bone marrow in short tau inversion recovery (STIR) images [11], which are typically used for imaging of bone marrow inflammation. The proposed method is potentially faster, easier and more robust than SPARCC and more importantly, eliminates the need of a trained reader. Another advantage of thresholding compared to SPARCC is that the former uses all images in the image set, while in the latter only a selection of slices is scored. Altogether, thresholding could be an alternative to SPARCC for quantification of subtle bone marrow inflammation in the spine and SI joints of patients with psoriatic arthritis. However, the validity of thresholding in this setting has not yet been tested.

This study aims to validate thresholding as a method suitable for accurate quantification of subtle bone marrow inflammation in patients with PsA and compare it with the SPARCC scoring system.

## Methods

### Patients

Patients diagnosed with PsA (*N* = 43) were recruited to the study, all being under optimal treatment at the time. Eligible patients were participating in a randomized clinical trial with high intensity interval training as intervention. Trial participants fulfilled the CASPAR-criteria for PsA, were between 18 and 65 years old and were able to exercise. Exclusion criteria were unstable PsA, unstable ischemic vascular disease, severe pulmonary disease, pregnancy, breastfeeding and drug or alcohol addictions. Two patients were excluded due to conditions that could influence the MR image analysis, one due to incidental findings (lymphoma) and one due to anomaly in the SI joints. Thus, 13 men with a mean age of 48 years (range: 30–64 years) and 28 women with a mean age of 48 years (range: 23–65 years) were included (*N* = 41). All patients have signed informed consent and the Norwegian Regional Committee for Medical and Health Research Ethics has approved the study. Patients were randomized into a control and an intervention group as part of a separate study. Effects of intervention are out of the scope of this study. Clinical evaluation at baseline, patient global assessment [mean ± standard deviation (SD): 42 ± 23 mm], disease activity score of 28 joints (mean ± SD: 2.9 ± 1.1), Bath ankylosing spondylitis disease activity index (mean ± SD: 3.4 ± 1.8), quality of life questionnaire, and high-sensitivity C-reactive protein (*hs-CRP*, median: 4.2 mg/L, range: 0.1 to 28.7 mg/L) provided patient health status.

### MRI

All patients underwent MRI examinations of the spine and the SI joints based on standardized protocols [12, 13]. Examinations were performed on two 1.5 T scanners (Scanner 1: Syngo MR B17 upgraded during the study to B19, Scanner 2: Syngo MR D13, Avanto, Siemens Healthcare, Germany). An inversion recovery based sequence (STIR) was used for the examination of the SI joints and the spine in two stations (Table 1). The protocol also included T1 and T2 weighted sequences for anatomical reference. American College of Radiology phantom tests were performed on both scanners as image quality control [14]. The effect of using different MR scanners was assessed with statistical analysis.

**Table 1:** Acquisition parameters

	Spine	SI joints
Orientation	Sagittal	Semi-coronal
TR (msec)	4250	3700
TE (msec)	51 (for lower spine) 52 (for upper spine)	52
TI (msec)	145	145
Slice thickness (mm)	4 (for lower spine) 3 (for upper spine)	4
Gap	10%	10%
Number of slices	Minimum of 16	15

*TR* time to recovery, *TE* time to echo, *TI* time to inversion

Clinical evaluation and MRI of the spine and SI joints were performed at one (*N* = 4), two (*N* = 20) and three time-points (*N* = 17). A total of 95 scans of the spine and the SI joints were acquired. Ten image sets of the spine were excluded from the analysis due to human error during the acquisition that resulted in altered protocol and different image weighting. A total of 85 scans of the spine and 95 scans of the SI joints were thus included in image analyses.

Acquisition parameters of short-tau inversion recovery (STIR) sequence used for the examination of the spine and the SI joints. Orientation, time to recovery (TR), time to echo (TE), time to inversion (TI), slice thickness, gap and number of slices are presented in the table. Images of the spine we acquired in two stations (lower spine and upper spine).

### SPARCC scoring

A rheumatologist (RST) trained for the SPARCC scoring methods, blindly scored the STIR images of the spine and the SI joints according to the SPARCC SI Joint and Spine Inflammation Indices [12, 13]. In short, for the spine, the six most abnormal disco-vertebral levels on the STIR sequence are selected. Three consecutive sagittal slices, that represent the most abnormal slices for each level, are chosen for scoring at that level. The total maximum SPARCC score is 108 for all six levels of the spine. In the SI joints, the six consecutive slices covering the cartilaginous part of the joints, which is the most relevant part of the SI joints when looking for inflammation, are scored. The total maximum SPARCC score is 72 for all six slices of SI joints. Cases with positive SPARCC scores were considered positive for the presence of bone marrow inflammatory edema, whereas cases with SPARCC score of 0 were considered negative.

For the spine, only a total SPARCC score per image set (*N* = 85) was provided, while for the SI joints both a total SPARCC score per image set (*N* = 95) and a SPARCC score for each chosen slice (*N* = 570) were available.

### Thresholding

#### Image pre-processing

Histogram-matching [15] is a histogram-based intensity normalization method that transforms the histogram of an image so that it is a match to the histogram of a reference image. Histogram-matching was performed to ensure that all image sets had the same overall brightness. All spinal MR images were histogram-matched to one reference spinal image and all MR images from SI joints were histogram-matched to one reference SI joint image. The function imhistmatch in MATLAB (MathWorks, Natick, MA, USA) was used.

#### Segmentation of bone marrow

Bone marrow of the sacrum and the iliac bones in the SI joints and vertebral bone marrow in the spine, excluding vascular and neural structures, were manually outlined using 3D Slicer (MIT Artificial Intelligence Lab, USA).

#### Volume of STIR hyper-intensity

All data processing was performed in Matlab R2016b (The MathWorks Inc., Natick, MA, 2000) using in-house scripts.

**Fig. 1:**
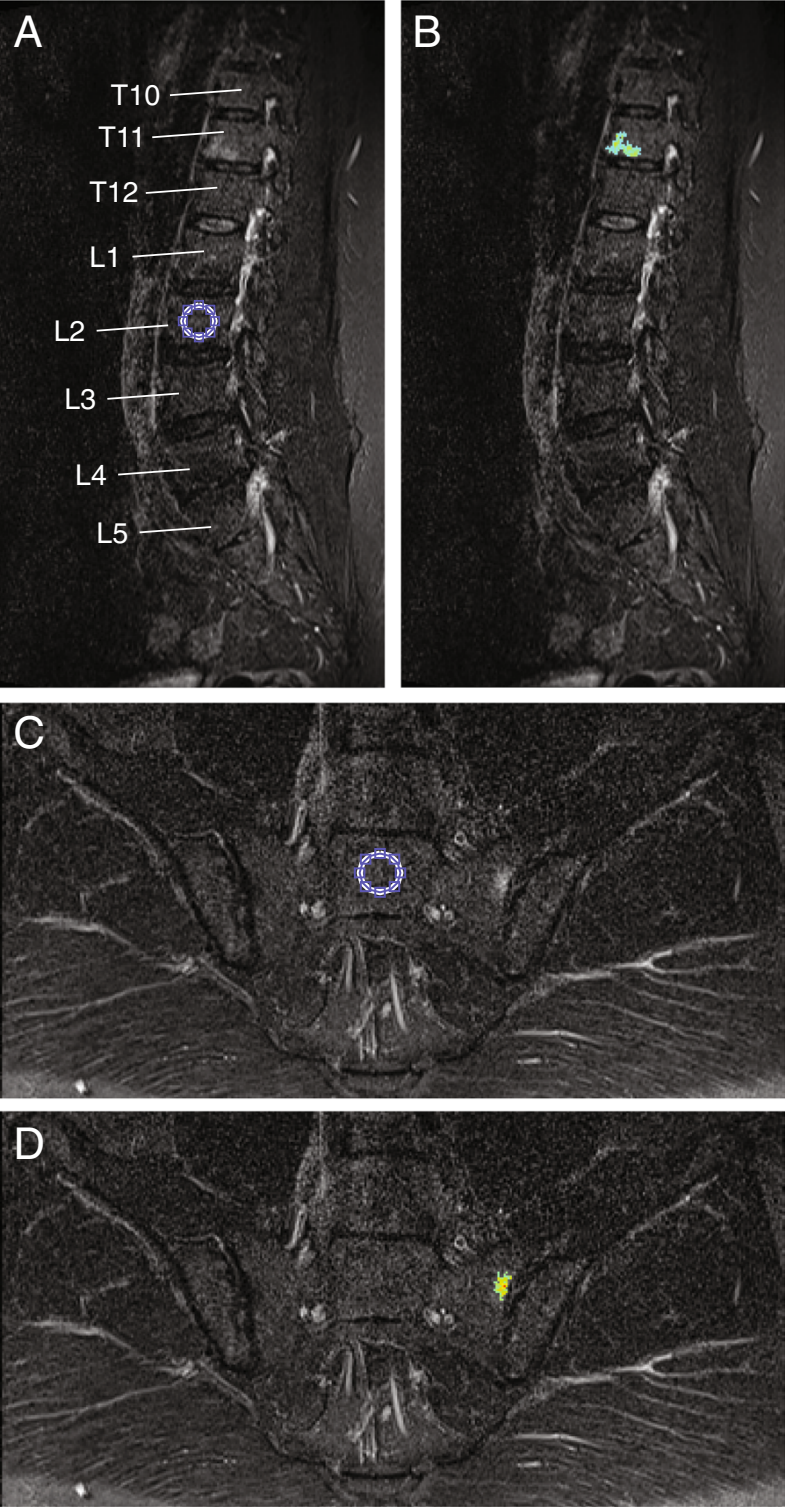
Example of placement of circular region of interest (ROI, ≥ 200 pixels) at the erector spinae muscles in short-tau inversion recovery (STIR) MR images of the spine (**a**) and gluteus maximus muscle in STIR MR images of the SI joints (**c**) of psoriatic arthritis patients for the normalization to signal from muscle tissue. For the selection of reference normal bone marrow signal as part of thresholding analysis, a circular ROI (≥ 200 pixels) was placed at a healthy vertebra in one slice of spinal images (**a**) and at the center of the first sacral vertebra in one slice in sacroiliac joint images (**c**). Example of thresholding of the volume of short-tau inversion recovery (STIR) hyper-intensity in a STIR MR image of the spine (**b**) corresponding to (**a**), and of sacroiliac joints (**d**) corresponding to (**c**). Vertebrae T10-L5 can be seen in (**a**) and (**b**). Inflammation was detected in T12. Iliac bones and sacrum are visible in (**c**) and (**d**)

**Fig. 2:**
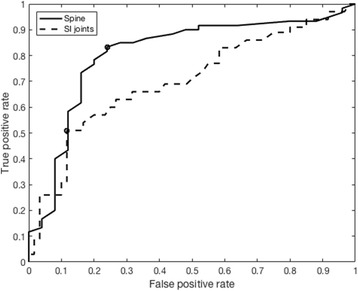
A receiver operating characteristic curve for the spine (continuous line) and the sacroiliac joints (dashed line) was plotted in order to define the optimal thresholds (shown in circle)

A signal intensity threshold consistent with inflammation was calculated from a circular ROI (≥ 200 pixels) at a healthy vertebra in one slice of the spinal image series and at the center of the first sacral vertebra in one slice of the SI joint image series (Fig. 1a, c). The criterion for choosing the ROI placement was the absence of bone marrow inflammatory edema. The mean signal intensity in this ROI was used as reference normal bone marrow signal intensity. A threshold was defined as the sum of the mean signal intensity in the reference normal bone marrow ROI and a percentage of the SD of signal intensity in that ROI. A receiver operating characteristic (ROC) curve was used to define the optimal threshold for the spine (area under curve [AUC] = 0.81) and the SI joints (AUC = 0.70) (Fig. 2). For the spine, the optimal threshold was defined as the sum of the mean signal intensity in the reference normal bone marrow ROI and 4.15 times the SD of signal intensity in that ROI. For the SI joints, the optimal threshold was defined as the sum of the mean signal intensity in the reference normal bone marrow ROI and 2.64 times the SD of signal intensity in that ROI.

All pixels with higher signal intensity than the threshold, consistent with inflammation [11], were selected and further used for the calculation of the volume of STIR hyper-intensity (*volume*_*hyper*_) in the vertebral bodies. All connected components (objects) in the resulting volumes that have fewer than 10 pixels were removed, as they were considered artefacts. *Volume*_*hyper*_ was acquired by adding the volumes of all hyper-intense pixels. The number of objects per image set, which represent different lesions, was calculated.

#### Relative signal intensities of STIR hyper-intense pixels

All hyper-intense pixels were normalized to the mean signal intensity of normal bone marrow [11]. The relative signal intensities of STIR hyper-intense pixels (*S*_*RelHyper*_) were calculated according to Eq. 1.

1 $$ {S}_{RelHyper}=\left({S}_{hyper}-\frac{\sum \limits_{i=1}^n{S}_{bone,i}}{n}\right)/\frac{\sum \limits_{i=1}^n{S}_{bone,i}}{n} $$

where *S*_*hyper*_ is the signal intensity value of the respective hyper-intense pixel, *S*_*bone*_ is the signal intensity of the pixel included in the reference normal bone marrow ROI and *n* represents the number of pixels in the reference normal bone marrow ROI [11]. The mean (*S*_*RelHyper*,  *mean*_), the median (*S*_*RelHyper*,  *median*_), 75-percentile (*S*_*RelHyper*,  75*perc*_) and 90-percentile (*S*_*RelHyper*,  90*perc*_) of *S*_*RelHyper*_ were calculated for all image sets.

### Statistical analysis

Spearman’s rank-order correlation between *SPARCC scores* and *volume*_*hyper*_, *S*_*RelHyper*,  *mean*_, number of objects per image set and *hs-CRP* and was calculated in IBM SPSS Statistics (IBM SPSS Statistics for Macintosh, Version 22.0).

Linear mixed-effects models (LMM) [16] were built in R 3.1.1 using the function *lme* from the ‘nlme’ package [17] employing the method of restricted maximum likelihood. LMM incorporate two types of effects: fixed, which are systematic and controlled, and random, which encompass unsystematic differences not accounted for by the fixed effects, e.g. variation between patients. The fixed effects are essentially different explanatory variables or classification factors whose relationship with the response variable is evaluated simultaneously. LMM models were built for data from both spine and SI joints separately, including the categorical fixed effects of *intervention group* (intervention or control), *time of scan* (time-point 1, 2 or 3), and *MR scanner* (machine 1 or 2) (without interaction terms). The continuous fixed effect of *SPARCC score* was also included, while the random effect was the *patient number* and the response variables were *volume*_*hyper*_, *S*_*RelHyper*,  *mean*_, *S*_*RelHyper*,  *median*_, *S*_*RelHyper*,  75*perc*_, *S*_*RelHyper*,  90*perc*_ or the number of objects per image set from thresholding. The latter were log10 transformed to comply with normality assumptions, confirmed by visual inspection of residual q-q plots and histograms.

We calculated sensitivity and specificity of thresholding compared to SPARCC from the proportion of patients identified with inflammatory lesions. Both for the spine (*N* = 85) and the SI joints (*N* = 95), the calculations were performed per image set including all the slices in each image set. In addition, for the SI joints, the calculations were performed per image set including only the six slices that were chosen for the SPARCC scoring method (N = 95) and per slice for the slices that were chosen for the SPARCC scoring method (*N* = 570).

## Results

### SPARCC

For the 85 image sets covering the spine, 60 were positive for inflammation using the SPARCC scoring method. For the 95 image sets covering the SI joints, 35 had a positive SPARCC score. Overall, 84 out of 570 slices of the SI joints were given a positive SPARCC score.

For the image sets with positive SPARCC scores, the mean score was 10.5 for the spine and 4.3 for the SI joints. Including all image sets, with positive or zero SPARCC scores, the mean SPARCC score for the spine was 7.4 ranging from 0 to 51 out of maximum possible score 108. The mean SPARCC score for the SI joints is 1.6, ranging from 0 to 17, out of a maximum possible score of 72.

### Thresholding

Thresholding revealed inflammatory lesions in 56 out of 85 image sets of the spine and 25 out of 95 image sets of the SI joints. In the analysis of SI joints, when including only the six slices that were chosen with the SPARCC method, 25 out of 95 image sets were found positive for the presence of inflammatory lesions. In total, 92 out of 570 slices of the SI joints showed inflammation when analyzed using thresholding.

For the image sets that had inflammatory lesions, mean *volume*_*hyper*_ was 2.92 cm^3^ and 2.77 cm^3^ for the spine and the SI joints, respectively. Including all image sets, with or without inflammatory lesions, mean *volume*_*hyper*_ was 1.92 cm^3^, ranging from 0 to 17.86 cm^3^, in the spine and 0.73 cm^3^, ranging from 0 to 19.04 cm^3^, in the SI joints.

The mean and the range of *volume*_*hyper*_, *S*_*RelHyper*,  *mean*_, *S*_*RelHyper*,  *median*_, *S*_*RelHyper*,  75*perc*_ and *S*_*RelHyper*,  90*perc*_ for the spine and the SI joints using all the slices are presented in Table 2. Examples of thresholding of the volume of STIR hyper-intensity in the SI joints and the spine are presented in Fig. 1.

**Table 2:** Volume of short-tau inversion recovery hyper-intense pixels and measures of lesion relative signal intensities

	Spine (*N* = 58)	SI joints (*N* = 36)
*volume*_*hyper*_ (cm^3^)	2.92 ± 3.86 (0.04–17.86)	2.77 ± 4.18 (0.03–19.04)
*S*_*RelHyper*, *mean*_	1.69 ± 0.12 (1.41–2.01)	0.66 ± 0.13 (0.45–0.92)
*S*_*RelHyper*, *median*_	1.72 ± 0.23 (1.31–2.26)	0.63 ± 0.23 (0.38–1.07)
*S*_*RelHyper*, 75*perc*_	2.09 ± 0.16 (1.79–2.26)	0.83 ± 0.23 (0.47–1.07)
*S*_*RelHyper*, 90*perc*_	2.24 ± 0.04 (2.05–2.26)	1.00 ± 0.14 (0.57–1.07)

*STIR* short-tau inversion recovery, *SI* sacroiliac, *volume*_*hyper*_ volume of STIR hyper-intensity, *S*_*RelHyper*_ relative signal intensities of STIR hyper-intense pixels, *75perc* 75-percentile, *90perc* 95-percentile

Volume of short-tau inversion recovery (STIR) hyper-intense pixels (*volume*_*hyper*_) and measures of lesion relative signal intensities; mean (*S*_*RelHyper*,  *mean*_), median (*S*_*RelHyper*,  *median*_), 75-percentile (*S*_*RelHyper*,  75*perc*_) and 90-percentile (*S*_*RelHyper*,  90*perc*_) of the relative signal intensities of STIR hyper-intense pixels for the spine and the SI joints calculated by thresholding. All values are given with standard deviations and parameter range in brackets.

### Statistics

Spearman’s rank-order correlation analysis revealed a significant positive correlation between SPARCC score and *volume*_*hyper*_ both for the spine (correlation coefficient: 0.74, *p* < 0.001) and the SI joints (correlation coefficient: 0.52, *p* < 0.001). SPARCC score did not correlate significantly with *hs-CRP*. Correlation coefficients calculated by Spearman’s rank-order correlation analysis are presented in Table 3.

**Table 3:** Spearman’s rank-order correlation

	SPARCC score			
	Spine		SI joints	
	Coefficient	*p*-value	Coefficient	*p*-value
*volume*_*hyper*_	0.74	< 0.001	0.52	< 0.001
*S*_*RelHyper*, *mean*_	0.67	< 0.001	0.47	< 0.001
Number of lesions	0.72	< 0.001	0.52	< 0.001
*hs-CRP*	−0.14	0.215	0.091	0.380

*SI* sacroiliac, *SPARCC* spondyloarthritis research consortium of Canada, *STIR* short-tau inversion recovery, *volume*_*hyper*_ volume of STIR hyper-intensity, *S*_*RelHyper*_ relative signal intensities of STIR hyper-intense pixels, *hs-CRP* high-sensitivity C-reactive protein

Results from multilevel LMMs to simultaneously assess the relationship between *volume*_*hyper*_, *S*_*RelHyper*,  *mean*_, *S*_*RelHyper*,  *median*_, *S*_*RelHyper*,  75*perc*_, *S*_Re*lHyper*,  90*perc*_ or the number of objects per image set and the fixed effects of SPARCC score, intervention group, time of scan and MR scanner are summarized in Table 4. A significant positive correlation between *volume*_*hyper*_ and SPARCC score was detected for spine (coefficient ± standard error: 0.11 ± 0.02, *p* < 0.001,) and SI joints (coefficient ± standard error: 0.31 ± 0.05, *p* < 0.001). The *intervention group*, *time of scan* (not shown) and the *MR scanner* were determined to not have a significant effect on the measurements by the thresholding method.

**Table 4:** Results from linear mixed-effects model

	Spine			SI joints		
	SPARCC score		MR scanner	SPARCC score		MR scanner
	Coefficient	*p*-value	*p*-value	Coefficient	*p*-value	*p*-value
*volume*_*hyper*_	0.11	< 0.001	0.467	0.31	< 0.001	0.804
*S*_*RelHyper*, *mean*_	0.09	0.001	0.347	0.25	< 0.001	0.597
*S*_*RelHyper*, *median*_	0.09	0.001	0.348	0.25	< 0.001	0.596
*S*_*RelHyper*, 75*perc*_	0.09	0.001	0.338	0.26	< 0.001	0.560
*S*_*RelHyper*, 90*perc*_	0.09	0.001	0.348	0.26	< 0.001	0.590
Number of lesions	0.13	< 0.001	0.424	0.37	< 0.001	0.672

*SI* sacroiliac, *SPARCC* spondyloarthritis research consortium of Canada, *STIR* short-tau inversion recovery, *volume*_*hyper*_ volume of STIR hyper-intensity, *S*_*RelHyper*_ relative signal intensities of STIR hyper-intense pixels, *75perc* 75-percentile, *90perc* 95-percentile

The two methods, SPARCC and thresholding, agreed on the absence of inflammatory activity in 19 out of 85 image sets of the spine, resulting in a sensitivity of 83% and a specificity of 76%. For the SI joints, the agreement was for 53 out of 95 image sets, resulting in a sensitivity of 51% and a specificity of 88%. When comparing the scores of each slice from the whole image set of SI joints, the two methods agreed on 434 slices out of 570 showing no inflammation, resulting in a sensitivity of 48% and a specificity of 89%.

Spearman’s rank-order correlation coefficients and *p*-values for the relationship of thresholding-derived metrics (volume, number of lesions and high-sensitivity C-reactive protein to spondyloarthritis research consortium of Canada.

Linear mixed-effects model (LMM) coefficients and *p*-values for the relationship of thresholding-derived metrics and number of lesions to spondyloarthritis research consortium of Canada (SPARCC) scores and MR scanner (scanner 1 or 2). The coefficients indicate how much *volume*_*hyper*_, *S*_*RelHyper*,  *mean*_, *S*_*RelHyper*,  *median*_, *S*_*RelHyper*,  75*perc*_ and number of lesions increase (positive coefficient) or decrease (negative coefficient) for every unit increase in the SPARCC score.

## Discussion

This study evaluates thresholding as a computer-aided method for quantification of subtle bone marrow inflammation in the spine and SI joints of PsA patients. Thresholding-derived metrics (*volume*_*hyper*_, *S*_*RelHyper*, *mean*_, *S*_*RelHyper*, *median*_, *S*_*RelHyper*, 75*perc*_, *S*_*RelHyper*, 90*perc*_ and number of objects per image set) correlate significantly with SPARCC scores both for the spine and the SI joints. However, the agreement on absence or presence of inflammation between the two methods was higher for the spine than for the SI joints, indicating that the proposed method of analysis performs better in the former. All metrics (mean, median, 75th-percentile and 90th-percentile) for the relative signal intensity of the hyper-intense lesions correlate with the same level of significance with the SPARCC scores. We therefore suggest that the *S*_*RelHyper*, *mean*_ can be used as a standard metric for relative signal hyper-intensity of inflammatory lesions.

To validate the use of the proposed method, we compared thresholding data to SPARCC scores for 85 image sets of the spine and 95 image sets of the SI joints from 41 PsA patients. In addition, for the 570 slices from SI joints, a slice-by-slice comparison was performed on results from the two methods. There was some disagreement between the two methods. The lesions that thresholding failed to detect in the spine (*N* = 10) had a mean SPARCC score of 3.8, while correctly identified lesions (*N* = 50) had a mean SPARCC score of 11.8. The disagreement was bigger for the SI joints, where lesions that thresholding failed to detect (*N* = 17) had a mean SPARCC score of 2.5, while correctly identified lesions (*N* = 18) had a mean SPARCC score of 5.9. Sensitivity and specificity measures show that thresholding analysis is more accurate in the spine. Spearman’s rank-order correlation analysis confirms higher correlation for the spine than the SI joints. Patients included in this study had little to no inflammation, especially in the SI joints, which may suggest that the method performs better in areas with higher inflammatory activity. Additionally, the examined anatomical structures in the spine are in the homogeneous image center of all slices, whereas the examined anatomical structures of the SI joints are more distant from the homogeneous image center, and also in varying distance through slices. This may affect the homogeneity of the acquired image. Areas that are closer to the coil appear more hyper-intense, resulting in slightly different signal intensities through an image. This issue could have been resolved using appropriate pre-processing. Anatomical differences may also contribute to lower lesion detectability in the SI joints. Additional pre-processing of the SI joint images could be used to correct for the inhomogeneities and signal intensity differences and improve the performance of thresholding in the SI joints.

Examinations were acquired using two MR scanners with different software platforms over the course of a year. During that time, one of the scanners underwent software upgrade. This should have no effect in the results of this study, and LMM also showed that different MR scanners used for imaging did not affect the measurements by the thresholding method.

The thresholding method presented here was first introduced in a previous study [11], where it was used to measure inflammatory changes in the SI joints of patients with lower back pain. However, in that study, the method was not compared to any clinical evaluation score and its validity was not tested. Additionally, the method was not tested for different threshold values to justify for the specific choice of threshold. In our study, the method is also applied in the spine.

One limitation of the thresholding method is that the ROIs of the bone marrow in the spine and the SI joints of the patients were drawn manually, in order to accurately exclude neural structures and blood vessels, but include possible inflammatory lesions. This presupposes a basic knowledge of the anatomy of SI joints. Fully automated methods for the selection of the sacrum and iliac bone ROIs should be explored. A fully automated method for the localization and segmentation of the vertebral units has been used in a previous study as part of a semi-automated framework for comparative visualization of inflammatory bone marrow lesions in MR images of the spine [18]. Combining fully automated segmentation of the spine and thresholding in such a setting could potentially assist in assessing radiological progression of patients with inflammatory lesions in the spine. Time required for SPARCC scoring depends on the experience of the reader, but also on how many lesions a patient has. A trained reader will need approximately 10 min for a patient without lesions and 30–40 min for a patient with many lesions. Time required for manual segmentation of bone marrow of a single image set is approximately 10 min. However, a fully automated segmentation of inflammation will reduce the reading time significantly and make thresholding a quantitative method feasible in the clinic.

A disadvantage of intensity-based methods for image analysis, such as thresholding, is that these methods are not able to differentiate between different pathologies that lead to increased signal intensities in the images, which is something a trained human can do easily. However, SPARCC scoring is used in patients who already have a diagnosis with a pre-investigative probability of having inflammation due to the primary diagnosis (psoriatic arthritis, spondyloarthritis, ankylosing spondylitis). Other approaches, including textural analysis, may be more beneficial in this instance. Another limitation of this study is the absence of a control group.

Overall, automatic thresholding is a novel method which performs relatively well at detecting inflammatory lesions in the spine of PsA patients, but more poorly in the SI joints. In addition to the presence or absence of inflammation, it provides volumetric information and allows localization of the lesions. The implementation of the method is generic enough to allow for application in the quantification of bone marrow inflammation in other types of spondyloarthritis. Fully automated implementation of the thresholding method should be explored.

## Conclusion

Thresholding allows quantification of subtle bone marrow inflammation in PsA patients with low SPARCC scores for inflammatory activity. The significant correlation for low inflammatory scores suggests that this method can provide reliable and sensitive quantitative measures for the presence of subtle inflammation in bone marrow. With further studies, automatic segmentation and technique optimization, it is possible that automatic thresholding may eventually be an alternative or supplement to SPARCC scoring.

## Abbreviations

75perc: 75-percentile
90perc: 95-percentile
AUC: Area under curve
*hs-CRP*: High-sensitivity C-reactive protein
LMM: Linear mixed-effects models
MRI: Magnetic resonance imaging
PsA: Psoriatic arthritis
ROC: Receiver operating characteristic
ROI: Region of interest
*S*_*bone*_: Signal intensity of pixel included in reference normal bone marrow ROI
SD: Standard deviation
*S*_*hyper*_: Signal intensity value of hyper-intense pixel
SI: Sacroiliac
SPARCC: Spondyloarthritis research consortium of Canada
*S*_*RelHyper*_: Relative signal intensities of STIR hyper-intense pixels
STIR: Short tau inversion recovery
*volume*_*hyper*_: Volume of STIR hyper-intensity
